# Fabrication of Soft Sensor Using Laser Processing Techniques: For the Alternative 3D Printing Process

**DOI:** 10.3390/ma12182955

**Published:** 2019-09-12

**Authors:** Myeongjoo Seo, Suwon Hwang, Taeseung Hwang, Junyeob Yeo

**Affiliations:** Novel Applied Nano Optics (NANO) Lab, Department of Physics, Kyungpook National University, 80 Daehak-ro, Bukgu, Daegu 41566, Korea

**Keywords:** laser, resin polymer, UV laser curing, laser nano welding, 3D printing process

## Abstract

Recently, the rapid prototyping process was actively studied in industry and academia. The rapid prototyping process has various advantages such as a rapid processing speed, high processing freedom, high efficiency, and eco-friendly process compared to the conventional etching process. However, in general, it is difficult to directly apply to the fabrication of electric devices, as the molding made by the rapid prototyping process is usually a nonconductive polymer. Even when a conductive material is used for the rapid prototyping process, the molding is made by a single material; thus, its application is limited. In this study, we introduce a simple alternative process for the fabrication of a soft sensor using laser processing techniques. The UV laser curing of polymer resin and laser welding of nanowires are conducted and analyzed. Through the laser processing techniques, we can easily fabricate soft sensors, which is considered an alternative 3D printing process for the fabrication of soft sensors.

## 1. Introduction

In the fourth industrial era, the importance of sensors that provide data for artificial intelligence in the Internet of Things (IoT) has progressively increased. Among the various sensor types, soft sensors for wearable devices [1,2,3,4,5,6] have received huge interest from both industry and research fields with the growth of the healthcare system market. Thus, the technology for the fabrication of soft sensors is also becoming more important.

In conventional fabrication processes for elastomer-based soft sensors [7,8,9,10], those processes usually require a high proficiency due to the complex process steps. In addition, there are disadvantages such as an inconsistent and monotonous sensor structure, which originate from manual manufacturing. Thus, various processing techniques have been introduced to the fabrication of soft sensors. 

The rapid prototyping (RP) process [11,12] is one of the most promising processes for the fabrication of soft sensors. The RP process has various advantages such as a consistent and diverse structure. In addition, the RP process enables the fabrication of complex and sophisticated three-dimensional (3D) structures. Thus, research on thinner and delicate high resolutions by the RP process was actively carried out, recently [13]. However, the RP process is not impeccably suitable for the fabrication of soft sensors, as materials available for the RP process are limited [14]; thus, it is difficult to pattern the polymer body and metal electrode, simultaneously.

Among various RP processes, the laser processing technique is one of the first methods employed and the most representative method in the RP process. Depending on the type of material used, the laser processing technique in the RP process is usually classified by the stereo-lithography (SLA) process for polymer resin [15], selective laser sintering (SLS) for metal nano particles [16], and selective laser melting (SLM) for metal powders [17]. In the case of the photo-curable resin, continuous liquid interface printing (CLIP) [14] and the two-photon polymerization (2PP) [18] process are also famous laser processing techniques in the RP process. These conventional RP processes using laser processing techniques have various advantages such as a fine resolution, complex structure, and high-accuracy structure. However, conventional RP processes using laser processing techniques are still not suitable for the fabrication of soft sensors due to the limitation of materials used, where it is difficult to pattern the polymer body and metal electrode simultaneously.

In this study, we introduce a simple alternative method for the fabrication of a soft sensor using laser processing techniques. The laser scanning system in this study is based on the SLA process. Thus, the polymer body is first fabricated by the ultraviolet (UV) laser curing process. Then, the spray coating process of silver nanowires (Ag NWs) is used for the conducting electrode of the sensor. In order to improve the electrical conductivity and mechanical properties of the sensor without thermal damage to the polymer substrate, the laser-induced nano welding (LINW) process [19,20,21] is applied to the sample as well. As the laser curing process and LINW process are conducted in one laser system, and the spray coating process can be applied to the arbitrarily shaped structure, we can simply fabricate an Ag NW-based soft sensor unlike the conventional RP process using laser processing techniques with the limitation of the material used.

## 2. Experimental Procedures and Optical Setup 

**Figure 1:**
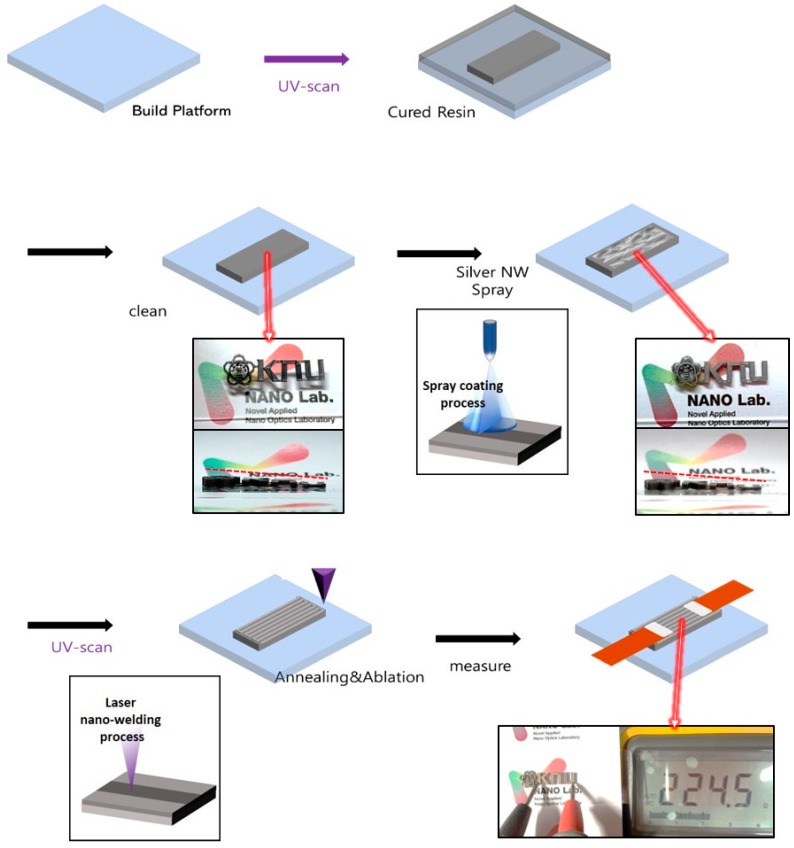
Schematic illustration for the fabrication of soft sensor using laser processing, and digital image of printed conducting 3D structure of “KNU” letters with different heights.

Figure 1 shows a schematic diagram of experimental procedure for the fabrication of soft sensor in this study. In addition, inset digital images are the printed conducting 3D structure of “KNU” letters with different heights by our laser scanning system. First, a photo-curable polymer resin (Form labs, flexible) is deposited onto the glass substrate. We use commercial polymer resin product ‘Flexible’ of Formlab without further purification. The polymer resin is a mixture of urethane acrylate (oligomer) and TPO (photoinitiator). Then, the deposited polymer resin is flattened by the doctor blading process and is cured by a UV laser successively. For the optimal conditions in the UV laser curing process, we examine various parameters such as laser power, laser scanning speed, laser spot size, and scanning hatch size. Then, uncured resin is washed out with IPA solution for 3 min.

To make a conducting electrode on the cured polymer substrate, Ag NWs (Novarial, A30UL) are deposited onto the polymer substrate using the spray coating process, as shown in the digital images of Figure 1. For the preparation of the Ag NW solution, 0.5 g of Ag NW solution (Novarial, A30UL) is diluted with 100 mL ethanol. In this step, the Ag NW percolation networks by the spray coating process form weak adhesion between the Ag NWs and substrate or between each deposited Ag NW. Thus, a post-annealing process is essentially required to improve the electrical conductivity and mechanical adhesion. There are several conventional post-annealing processes such as a convection oven, furnace, and hot plate; however, those processes are usually not suitable for the polymer substrate due to the low melting temperature of the polymer. Therefore, in the final step, the LINW process is applied to the Ag NWs for the enhancement of electrical conductivity and mechanical properties (adhesion enhancement). After deposition of Ag NWs, copper tapes and silver paste (active Ag NW sensing part at 2.5 cm × 1.5 cm) are attached to the sample for the measurement of electrical conductivity. Through this whole process, we can simply fabricate a Ag NW-based soft sensor, which shows the possibility of an alternative 3D printing technique for the fabrication of soft sensors.

For the measurement of the electrical and mechanical characteristics of the Ag NW-based soft sensor, a multimeter (34401A, Agilent, Santa Clara, CA, USA) and lab-made test machine including a linear stage (X-LHM150A, Zaber Technologies, Vancouver, BC, Canada) are used. Thus, the mechanical reliability (bending and strain fatigue test) of the Ag NW-based soft sensor is evaluated by the multimeter and lab-made test machine.

**Figure 2:**
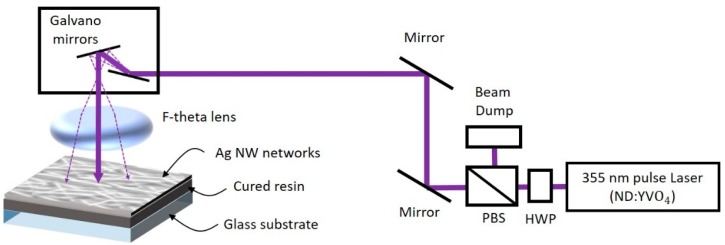
Optical apparatus layout of this study.

Figure 2 shows the optical setup layout. In this study, the laser scanning system is based on a modified SLA process. For the 2D plane laser patterning, a Galvano mirror (HurryScan II 10, Scanlab, Puchheim, Germany) and f-theta lens (f = 103 mm) are combined. Then, a motorized 3D stage (X-XYZ-LSQ150B-K0060-SQ3, Zaber Technologies) in the sample plane is added to complete the 3D printing system. For the curing of the resin polymer, a 355 nm nanosecond pulse laser (Poplar-355-3A, Huaray, Wuhan, China) is used. The laser power is adjusted by combining a polarized beam splitter (PBS) and half-wave plate (HWP). 

## 3. Results and Discussions

**Figure 3:**
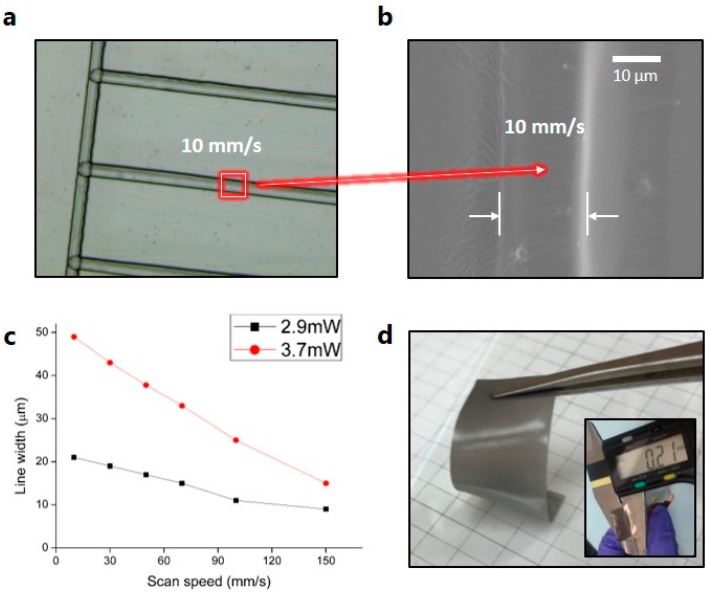
(**a**) Optical microscopy image of cured resin by UV laser curing process. (**b**) SEM image of cured resin by UV laser curing process. (**c**) Linewidths of cured resin with different scanning speeds at two fixed laser powers. (**d**) Digital image of fabricated polymer substrate by UV laser curing process.

On the sample at the focal plane of the f-theta lens, the liquid photo-curable resin polymer absorbs laser energy and the resin is then cured/solidified. Figure 3a,b show an optical image and SEM image of the patterned line on the glass substrate after curing the resin polymer by the UV laser curing process, respectively. In addition, Figure 3c shows the measured line width of the cured resin with different scanning speeds at two fixed laser powers (2.9 and 3.7 mW). It is confirmed that the line width of the cured resin is directly related to the laser power and the scanning speed of the focused laser spot. In addition, Figure 3c shows the inversely proportional relationship between the line width of the cured resin and the laser scanning speed.

Theoretically, the beam waist radius (1/e^2^) of the focused laser in this optical system is estimated to be ~5 μm. However, resin polymer curing by the UV laser can be realized near the beam waist, as the laser beam is Gaussian. Thus, the optimized line width of the cured resin polymer is about 10 μm, which is bigger than the theoretical beam waist.

Meanwhile, the height of the cured resin is dependent on the doctor blading process in this study. For the fabrication of a robust soft sensor, we fixed the height of the cured resin to around 200 μm, as shown in Figure 3d. In addition, we fixed the polymer sample size at 5 cm × 1.5 cm and active Ag NW sensing part at 2.5 cm × 1.5 cm for further study.

**Figure 4:**
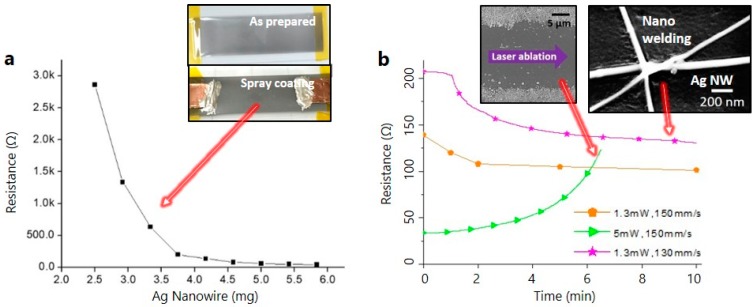
(**a**) Resistances of deposited silver nanowire (Ag NW) layer with different concentrations of Ag NWs. Inset picture shows digital images before and after spray-coating the Ag NWs. (**b**) Resistances of Ag NW layer during laser-induced nano-welding process with different laser powers and scanning speeds. Inset pictures show magnified SEM images of Ag NW layer after laser-induced nano-welding and laser ablation processes.

To make a conducting electrode of the soft sensor, Ag NWs are deposited by the spray coating process onto the cured resin polymer substrate. After deposition of Ag NWs by the spray coating process, the color of the sample surface slightly changed, as shown in the inset picture of Figure 4a. As the Ag NW percolation network is formed on the cured resin polymer substrate by the spray coating process, Figure 4a shows that the measured resistance decreases as the amount of Ag NWs increases. 

In this study, as mentioned in the experimental procedures, the LINW process is applied to the Ag NW percolation networks as a post-annealing process. With adjustment of laser power, Ag NWs can be annealed or ablated, as shown in the magnified SEM pictures of the inset of Figure 4b. When the laser irradiates the Ag NWs, localized surface plasmon resonance (LSPR) is induced on the surface of Ag NWs [22,23,24]. Thus, the induced LSPR generates localized thermal heating at the junction of Ag NW percolation networks due to the electromagnetic field enhancement. This is the reason why Ag NWs are slightly melted or well-ablated by the LINW process at the junction of Ag NW percolation networks, as shown in the magnified SEM pictures of the inset of Figure 4b, which is similar to previous studies concerning the laser processing of Ag NWs [19]. Thus, through the LINW process, the mechanical properties (adhesion enhancement), as well as the electrical conductivity, of Ag NW percolation networks are easily improved without polymer substrate damages.

As the LINW process is applied between both copper electrodes (length between both copper electrodes is about 2.5 cm) for the measurement of electrical conductivity, the resistance is gradually changed, as shown in Figure 4b. The resistance drops during the LINW process with laser scanning speeds of 130 and 150 mm/s at a moderate laser power of 1.3 mW and scanning pitch size of 10 μm. Figure 4b shows ~25% electrical conductivity improvements of Ag NW percolation networks within 10 min (from 207 to 153 Ω, from 139 to 101 Ω). Meanwhile, a high laser power of 5 mW can ablate and destroy the Ag NW percolation networks, as shown in the left inset SEM picture of Figure 4b. Figure 4b shows that the resistance increases gradually during the LINW process at a high laser power of 5 mW. Even though the electrical conductivity is not significantly changed compared to the previous results [20,21], the LINW process helps to obviously enhance the mechanical properties (adhesion enhancement between Ag NWs and substrate or between each deposited Ag NW), as well as the electrical conductivity. In this study, we fixed the laser power and laser scanning speed at 1.3 mW and 130 mm/s for the post annealing of Ag NW percolation networks, respectively.

**Figure 5:**
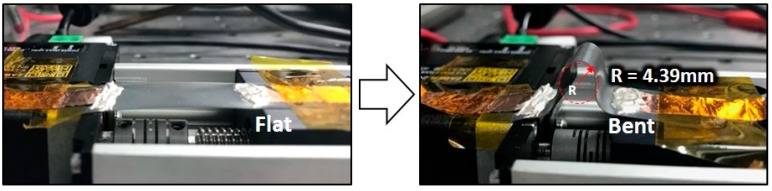
Digital images of cyclic bending/stretching test experiment at flat (left) and bent (right) state with bending radius of 4.39 mm.

The mechanical reliability of the Ag NW-based soft sensor is evaluated with a bending and strain fatigue cyclic test. As shown in Figure 5, a lab-made test machine is used to evaluate the mechanical reliability of the Ag NW-based soft sensor. The bending radius is calculated by Equation (1) [25]:

(1) $$\mathrm{Bending}\;\mathrm{radius}\;(r)=\frac{L}{2\pi \sqrt{\frac{dL}{L}-\frac{\pi^{2}h_{s}^{2}}{12L^{2}}}},$$

where *L*, *dL*/*L*, and *h_s_* represent the initial length of the substrate, ratio of changed length, and substrate thickness, respectively. The bending and strain fatigue tests are conducted at a frequency of 0.5 Hz for a duration of 1000 and 60 cycles, respectively. 

**Figure 6:**
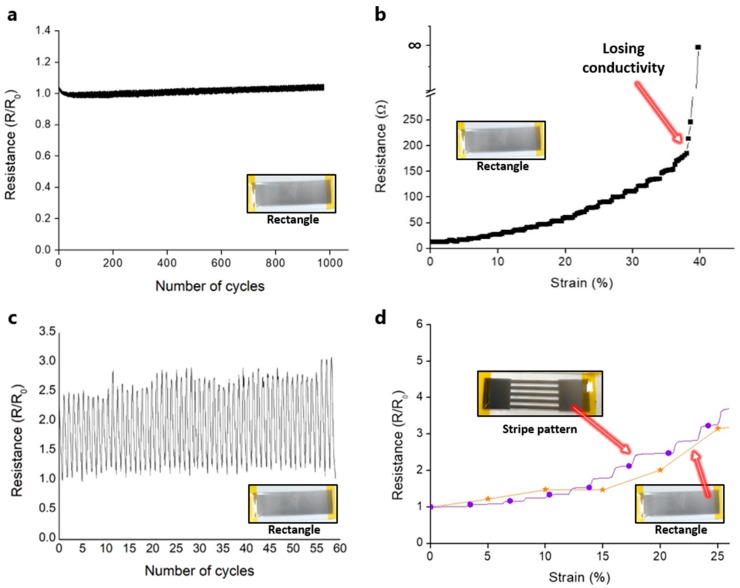
(**a**) Bending fatigue test at the number of bending cycles for the Ag NW-based soft sensor. (**b**) The resistance at the different applied strains for the Ag NW-based soft sensor. (**c**) Strain fatigue test at the number of strain cycles for the Ag NW-based soft sensor. (**d**) The strain-dependent electrical characteristics of the Ag NW-based rectangular and stripe-pattern soft sensor.

Figure 6a shows the resistance ratio (R/R_0_) of the Ag NW-based soft sensor with different bending cycles. The initial resistance (R) of the sample and bending radius (*r*) in the bending test are 39 Ω and 4.39 mm, respectively. As shown in Figure 6a, the resistance ratio does not show noticeable changes through the bending fatigue test during 1000 cycles.

Figure 6b shows the strain-dependent electrical characteristics of the Ag NW-based soft sensor. The resistance (R_0_ = 12 Ω) increases almost proportionally along the applied strain below 25% strain while the resistance changes show a slightly stepped variation above 25% strain. In addition, the resistance goes to infinity over 40% strain due to the broken networks of Ag NWs. Thus, the applied strain is fixed at 25% for the further strain fatigue test. 

Figure 6c shows the resistance ratio (R/R_0_, R_0_ = 20.8 Ω) of the Ag NW-based soft sensor with different strain cycles. The resistance ratio varies along with the applied strain, while the variation width of the resistance ratio is slightly increased during the cyclic test. Generally, the initial resistance in the NW-based soft sensor is not fully recovered during the cyclic test due to aging problems of NW networks [26,27] and interconnection problems between NWs [28]. In addition, the UV-cured resin polymer is usually stiff and rigid after the UV curing process. Meanwhile, the cured resin substrate in this study is somewhat ductile and soft due to the thin thickness (~200 μm) of the cured resin substrate. Thus, the measured resistance ratio in the strain test does not stabilize perfectly during the cyclic test, yet it shows good flexibility and the possibility as a strain sensor.

Meanwhile, the polymer substrate is fabricated by the SLA-based UV laser scanning technique in this study. Thus, we can make an arbitrary shape of the polymer substrate through the UV laser scanning system and apply it to easily fabricate two types (rectangular and stripe) of soft sensors. Figure 6d shows the strain-dependent electrical characteristics of the Ag NW-based rectangular and stripe-pattern soft sensor. The gauge factor (GF) is calculated by Equation (2):

(2) $$\mathrm{Gauge}\;\mathrm{factor}\;(\mathrm{GF})=\frac{\Delta R/R}{\Delta L/L},$$

where *L*, Δ*L*, *R*, and Δ*R* represent the initial length of the substrate, change in length, unstrained initial resistance, and change in strained resistance due to strain, respectively. The initial resistance and measured GF of the rectangular soft sensor are 20.8 Ω and 9.65, respectively. In addition, the initial resistance and measured GF of the stripe-pattern soft sensor are 42 Ω and 13, respectively. Even though the resistance changes of the stripe-pattern soft sensor show a stepped variation within 25% strain, the stripe-pattern soft sensor shows a higher GF than the rectangular soft sensor, which means that the GF and performance of the soft sensor can be affected by the geometrical shape of the sensor. As the Ag NW spray coating process can be applied to any 3D structure to construct a conducting electrode, we can easily fabricate various arbitrarily shaped soft sensors. Therefore, we expect that our approaches will be suitable for the fabrication of soft sensors in the future rather than manual manufacturing, which has disadvantages such as the inconsistency and monotony of the sensor structure.

## 4. Conclusions

In this research, we demonstrate the fabrication of a soft sensor using laser processing techniques and a spray coating process. Through the SLA and LINW process, we can successfully fabricate the polymer body and metal electrode pattern with the same optical apparatus without additional complex processing steps. In addition, a metal electrode is easily deposited by the spray coating process of Ag NWs. Then, the LINW process improves the electrical conductivity, to about 25%, and the mechanical adhesion of the soft sensor. Due to the flexibility and ductility of Ag NWs, the fabricated soft sensor operates successfully under bending and strain. The rectangular shaped sensor and stripe-pattern soft sensor can operate up to a 40% stretch, and the gauge factors are calculated as 9.65 and 13, respectively. Moreover, the 1000-cycle bending test shows stable operation with the 4.39 mm bending radius.

The spray coating process of the Ag NW-combined 3D printing technology for the fabrication of soft sensors has not been reported yet up to now. Thus, we suggest these combined laser processing techniques (SLA and LINW) and spray coating as an alternative 3D printing process for the fabrication of soft sensors, as the LINW process can be applied to the SLA optical setup without additional equipment. Especially, with the simple spray coating process, we expect our approach will be a new concept as an alternative 3D printing process for the patterning of the polymer body and metal electrode simultaneously. The spray coating process of Ag NWs is key to overcoming current 3D printing technology. It can easily cover every surface of 3D structures that have different heights and deposit conducting layers without etching processes. Furthermore, conventional SLA 3D printing devices can be easily changed to alternative 3D printing based on our techniques by adding only a spray module. Therefore, we suggest our laser processing techniques and spray coating technique as an alternative 3D printing process for the fabrication of soft sensors.
